# Pediatric Inflammatory Bowel Disease in Romania: The First Epidemiological Study of the North-West Region (2000–2020)

**DOI:** 10.3390/children12040403

**Published:** 2025-03-22

**Authors:** Georgia Valentina Tartamus (Tita), Daniela Elena Serban, Cristina Rebeca Fogas, Marcel Vasile Tantau

**Affiliations:** 1 3rd Medical Discipline, Department of Internal Medicine, “Iuliu Hatieganu” University of Medicine and Pharmacy, 400012 Cluj-Napoca, Romania; georgia.tartamus@umfcluj.ro (G.V.T.); fogas.cristina.rebeca@elearn.umfcluj.ro (C.R.F.); 2 Department of Mother and Child, 2nd Clinic of Pediatrics, Emergency Clinical Hospital for Children, “Iuliu Hatieganu” University of Medicine and Pharmacy, 400177 Cluj-Napoca, Romania; 3 Department of Internal Medicine and Gastroenterology, “Prof. Dr. Octavian Fodor”, Regional Institute of Gastroenterology and Hepatology, “Iuliu Hatieganu” University of Medicine and Pharmacy, 400162 Cluj-Napoca, Romania; marcel.tantau@umfcluj.ro

**Keywords:** inflammatory bowel disease, Crohn’s disease, ulcerative colitis, incidence, temporal trends

## Abstract

**Background**: Inflammatory bowel disease (IBD) represents a group of disorders with increasing incidence in the pediatric population worldwide. There are limited data on pediatric IBD (pIBD) epidemiology in Eastern Europe and none so far from Romania. The aim of our study was to evaluate incidence rates and time trends for pIBD in the north-west region of Romania and to compare them with results from studies on the same topic published worldwide. **Methods**: We performed a retrospective study of patients under 18 years of age diagnosed with pIBD in the time frame between 1 January 2000 and 31 December 2020 at the Emergency Clinical Hospital for Children in Cluj-Napoca. Age-adjusted incidence rates, annual percentage change (APC), average annual percentage chance (AAPC) and their corresponding 95% confidence intervals (CIs) were calculated for pIBD, Crohn’s disease (CD) and ulcerative colitis (UC). Temporal trends were plotted using Joinpoint regression. **Results**: Ninety-four patients were identified. For the entire studied period, the incidence rate for pIBD was 0.79/10^5^ (±0.74), 0.4/10^5^ for CD (±0.42) and 0.34/10^5^ for UC (±0.4). Time trends for incidence were positive, but statistical significance was found only for pIBD and CD. The APC observed for pIBD, CD and UC was 12 (95% CI: 6.5–17.7), 13.1 (95% CI: 8–18.5) and 5.67 (95% CI: 1.5–13.4), respectively. Comparison to other similar studies placed Romania among the countries with the lowest incidence of pIBD. **Conclusions**: Although pIBD incidence in our region appears to be low, there has been an important and significant increase in the incidence of overall pIBD and especially CD.

## 1. Introduction

Inflammatory bowel disease (IBD) represents a group of disorders characterized by chronic idiopathic inflammation of the gastrointestinal tract, sometimes with extraintestinal manifestations, including Crohn’s disease (CD), ulcerative colitis (UC) and unclassified IBD (IBD-U), the first two types being the most prevalent. IBD is considered a multifactorial disorder, where genetic predisposition, epigenetic modifications, environmental factors, alterations in gut microbiota and immune dysregulation interact to play a role in the onset and progression of the disease [1]. The treatment of IBD aims to achieve and maintain remission and promote good quality of life and growth without disability [2]. The approach is multidisciplinary, including nutritional interventions, pharmacological therapies (aminosalicylates, corticosteroids, immunomodulators, biologics and small molecules) and surgical management [3,4,5,6,7,8,9]. Dietary interventions in IBD aim to reduce intestinal inflammation, modulate gut microbiota composition, support mucosal healing and improve nutritional status, serving as both primary and adjunctive therapies in disease management. Multiple dietary approaches have been trialed in IBD including exclusive enteral nutrition (EEN), the Mediterranean diet, the low-FODMAP diet, the CD Exclusion Diet (CDED), the UC Exclusion Diet (UCED) and the specific carbohydrate diet (SCD), with varying levels of evidence for their effectiveness [10].

Being a chronic relapsing disease, the rise in incidence over time has resulted in a high prevalence and a significant burden for healthcare systems [11]. IBD tends to be more frequent in the early-adult and adolescent age groups, with almost one-quarter of patients having their first symptoms before the age of 18 years [12,13].

Pediatric IBD (pIBD) displays particularities in terms of disease manifestations, with more extensive involvement and rapid early progression as well as more complicated behavior. Also, children with IBD are at risk of growth impairment, delayed puberty, bone structure and function damage and psycho-social difficulties [14,15,16].

In the second half of the 20th century, IBD had a rising incidence in Westernized nations (Europe, North America, Australia, New Zealand). At first, epidemiological studies showed a UC to CD ratio in favor of UC, but later, the incidence of CD caught up and even exceeded that of UC in some regions. By the beginning of the 21th century, IBD became a global disease in newly industrialized countries, facing an increase in the number of cases, while the Westernized world experienced a stabilization of incidence [11,17]. Since the 1990s, an upward trend in pIBD incidence has been seen. Recent epidemiological studies focusing on pIBD have reported an increasing incidence across the world at least in one age or in one disease-specific group, but signs of plateauing incidence have also been seen in some regions (England, Slovenia) [18,19]. In Europe, a north to south gradient was observed, with higher incidence in Northern Europe than in other regions [20]. Still, the global epidemiology of pIBD remains a knowledge gap due to the scarce data from under-developed and developing countries.

The aim of our study was to provide the first comprehensive epidemiological analysis of pIBD in Romania, focusing on incidence in the north-west region over a 21-year period (2000–2020). In addition, this study compared our findings with published epidemiological trends worldwide, offering insights into regional differences and contributing to the global understanding of pIBD.

## 2. Materials and Methods

### 2.1. Study Design

We conducted a retrospective observational study of the pIBD patients diagnosed between January 2000 and December 2020 residing in the north-west region of Romania. The region includes the 6 following counties: Cluj, Sălaj, Bistrița-Năsăud, Maramureș, Satu Mare and Bihor. The study was approved by the Ethics Committee of “Iuliu Hatieganu” University of Medicine and Pharmacy, Cluj-Napoca, Romania.

### 2.2. Patients

The study cohort included all patients diagnosed with pIBD at the Emergency Clinical Hospital for Children (ECHC) in Cluj-Napoca, a high-volume tertiary care pediatric academic medical center. Patients over 18 years old at the time of the diagnosis were excluded. The diagnosis of pIBD was established using the revised PORTO criteria of ESPGHAN, requiring a combination of clinical evaluation, laboratory markers, endoscopic and histopathologic findings and imaging techniques, as well as the exclusion of enteric infections. Clinically, pIBD presents with persistent gastrointestinal symptoms such as chronic diarrhea, abdominal pain, rectal bleeding, weight loss and growth impairment, often accompanied by extraintestinal manifestations. Laboratory markers include elevated C-reactive protein, erythrocyte sedimentation rate and fecal calprotectin, the latter serving as a key non-invasive screening tool. Endoscopic evaluation, including lower and upper gastrointestinal endoscopy with biopsies, is mandatory to differentiate disease subtypes. In cases confined to the colon, where features overlap, a diagnosis of IBD-U is considered. For suspected CD with small bowel involvement, magnetic resonance enterography, intestinal ultrasound and wireless capsule endoscopy are the diagnostic methods of choice [21].

### 2.3. Methods

Data regarding patients were collected from the medical files and/or from the hospital’s electronic database. The following medical and demographic information was retrieved: IBD subtype, county of origin, living setting, sex, age at diagnosis, comorbidities, IBD family history and year of diagnosis. Patients were divided into IBD subtypes: UC, CD and IBD-U. We obtained the information about the region’s population size for each year of the study period from the Romanian National Statistics Institute (TEMPO online database) [22]. We conducted a literature search of PubMed and Web of Science databases from inception through December 2022 to identify national or regional studies assessing incidence rates and incidence temporal trends for pIBD, pediatric CD and pediatric UC within a timeframe similar to that in our study. We compared the findings from the literature search with our study results and presented them in charts.

### 2.4. Statistical Analysis

Incidence rate per 100,000 inhabitants (crude rate) was calculated through the division of new pIBD cases by the number of inhabitants of a given year multiplied by 100,000. The age-adjusted rate (AAR) was calculated using the direct standardization method, based on the 2013 European Standard Population, used as a reference [23]. For the analysis of temporal trends for incidence, Joinpoint regression was used, allowing us to test whether the incidence was better explained by a single trend or by the existence of multiple trend segments. Annual percentage change (APC), average annual percentage chance (AAPC) and their corresponding 95% confidence intervals (CIs) were calculated. All hypotheses were tested at the 0.05 significance level. All of the above analyses were performed in the Joinpoint Regression Program, Version 4.9.0.1 (Statistical Research and Applications Branch, National Cancer Institute, Bethesda, MD, USA). Quantitative data were summarized using median and interquartile range (IQR) or mean and standard deviation, according to the type of distribution. For qualitative data, count and relative frequency (%) were employed. Statistical analyses were performed with Microsoft Excel (Microsoft Corporation, Redmond, WA, USA) functions.

## 3. Results

During the study period, 94 children under the age of 18 years living in the north-west part of Romania were diagnosed with IBD. UC was diagnosed in 41 patients (43.6%), CD in 48 patients (51%) and IBD-U in 5 of them (5.4%). Regarding the counties of origin, Cluj had the highest representation (36.1%), followed by Maramureș (24.4%), Bihor (11.7%), Satu Mare (10.6%), Sălaj and Bistrița-Năsăud, each with 8.5%. Almost three-quarters of the patients (73.4%) were from the urban areas. Among the cases, 55 (58.5%) were males and 39 (41.5%) females. The median age at diagnosis for pIBD was 14 years (IQR 4.7), with a minimum age of 3.5 years and a maximum of 17.9 years, with both cases classified as UC. For UC patients, the median age of diagnosis was 13.1 years (IQR 5.3); for CD, it was 14.5 years (IQR 5.0); and for IBD-U, it was 11 years (IQR 5.4). Table 1 provides information about the distribution of patients according to the year of diagnosis, age group, type of disease and sex.

In this study cohort, anemia was the most common comorbidity, affecting 12.7% of patients, followed by articular involvement (oligoarthritis, sacroiliitis) and hepato-biliary disease (primary sclerosing cholangitis, autoimmune hepatitis) in 6.3% of cases; skin involvement (erythema nodosum, skin vasculitis, ectodermal dysplasia) and oral aphthous lesions were observed in 2.1% and 1% of cases, respectively. Additionally, allergic manifestations (rhinoconjunctivitis, asthma) were identified in 6.3% of cases and autoimmune thyroiditis in 1%. Immunological alterations were also noted, with low levels of immunoglobulin type A (3.1%), high levels of immunoglobulin type A (5.3%), low levels of immunoglobulin type G (2.1%) and high levels of immunoglobulin type G (3.1%). Three patients (3.1%) with pIBD (one with CD and two with UC) had a positive family history for IBD (first-degree relatives).

**Figure 1:**
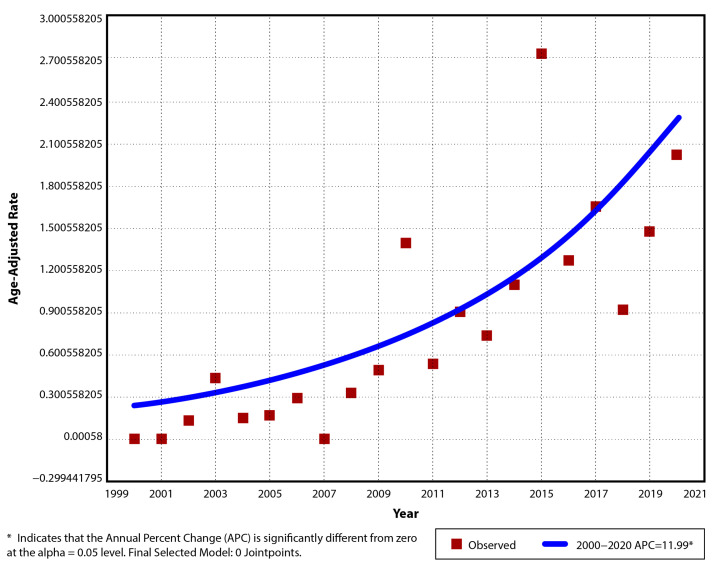
Temporal trend for pIBD age-adjusted incidence rates for period 2000–2020.

The annual incidence of pIBD in the period 2000–2020 was 0/10^5^ for the years 2000, 2001 and 2007, and 2.13/10^5^ (AAR 2.01/10^5^, 95% CI: 0.81–3.21) for 2020, with a maximum of 2.86/10^5^ (AAR 2.73/10^5^, 95% CI: 1.34–4.12) in 2015. Between 2000 and 2010, the mean incidence was 0.3/10^5^ (±0.4), and from 2011 to 2020, it was 1.33/10^5^ (±0.66), with an average of 0.79/10^5^ (±0.74) for the entire period (Table 2). The suggested model had only one trend segment. The positive linear trend over that time period was significant (*p* < 0.001), with an increase of 12% per year in the incidence rate (APC 12, AAPC 12, 95% CI: 6.5–17.7) (Figure 1).

**Figure 2:**
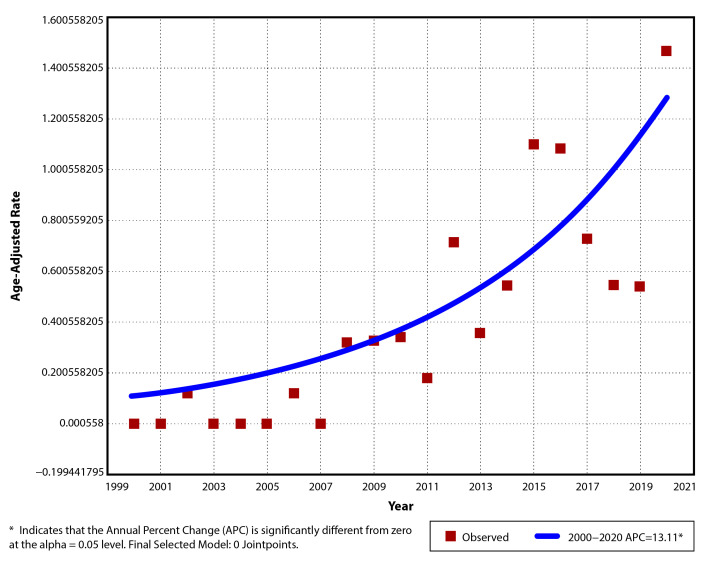
Temporal trend for pediatric CD age-adjusted incidence rates for period 2000–2020.

For CD, the annual incidence in the studied period varied from 0/10^5^ in 6 of the first 8 years (exception 2002 and 2006) to a maximum of 1.55/10^5^ (AAR 1.47/10^5^, 95% CI: 0.45–2.49) in 2020. The average incidence of CD cases diagnosed between 2000 and 2020 was 0.4/10^5^ (±0.42), with a mean of 0.11/10^5^ (±0.15) in the first 11 years and 0.72/10^5^ (±0.39) for the last decade (Table 3). A significant upward trend was observed (*p* < 0.001), with an increase of 13.1% per year in the incidence rate (APC 13.1, AAPC 13.1, 95% CI: 8–18.5). No joinpoint was suggested for this trend (Figure 2).

**Figure 3:**
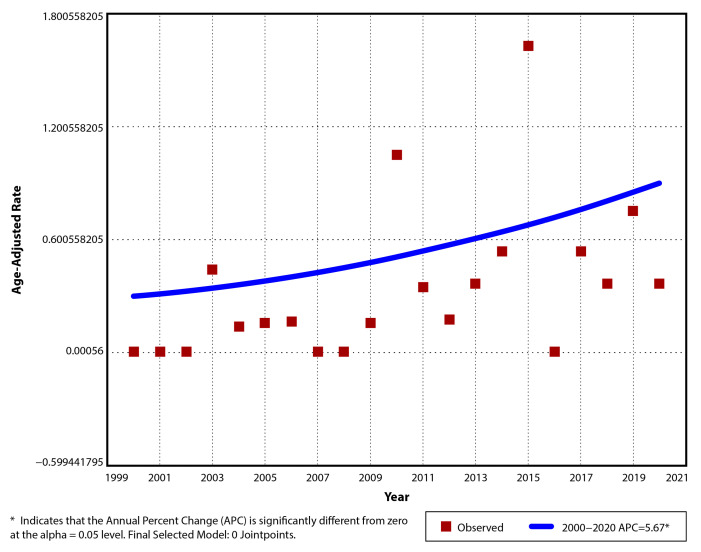
Temporal trend for pediatric UC age-adjusted incidence rates for period 2000–2020.

The incidence of UC per year between 2000 and 2020 was 0/10^5^ in the first 3 years (2000, 2001, 2002) and 0.39/10^5^ (AAR 0.37/10^5^, 95% CI: −0.14–0.88) in the last year (2020) and had a maximum of 1.71/10^5^ (AAR 1.63/10^5^, 95% CI: 0.57–2.69) in 2015. The mean incidence of UC was 0.34/10^5^ (±0.4) for the entire study period, 0.19/10^5^ (±0.3) between 2000 and 2010, and 0.51/10^5^ (±0.44) between 2011 and 2020 (Table 4). The observed trend was ascending, with an APC and AAPC of 5.67 (95% CI: −1.5–13.4), but it did not reach significance (*p* = 0.11). A single segment trend was suggested for this situation (Figure 3).

IBD-U was diagnosed from 2016 with a mean incidence of 0.045/10^5^ (±0.1).

Between the two studied periods (from 2000 to 2010 and from 2011 to 2020), we observed no difference in sex distribution for pIBD (*p* = 0.38).

**Figure 4:**
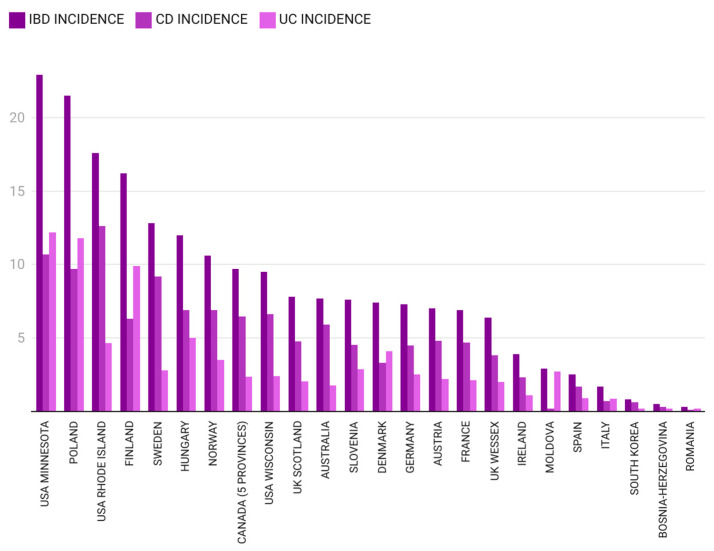
Pediatric IBD incidence in different regions of the world between 1995 and 2010 [12,24,25,26,27,28,29,30,31,32,33,34,35,36,37,38,39,40,41,42,43,44,45,46] (the authors summed CD and UC incidence for Bosnia–Herzegovina and calculated the mean IBD incidence for Germany).

**Figure 5:**
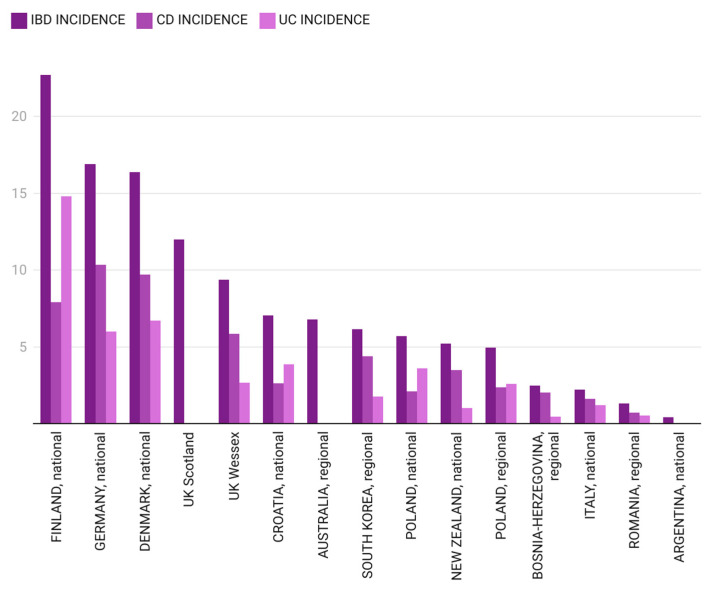
Pediatric IBD incidence in different regions of the world between 2009 and 2020 [12,25,27,33,36,40,44,47,48,49,50,51,52,53,54] (for South Korea, the authors calculated the mean incidence).

A comparative analysis of incidence rates for pIBD, CD and UC in different regions of the world for two specific time frames (1995–2010 and 2009–2020) is presented in Figure 4 and Figure 5.

## 4. Discussion

To the best of our knowledge, the present study is the first to perform an epidemiologic analysis of pIBD in Romanian children (under 18 years old) over a period of 21 years. We observed for the entire study period a mean age-adjusted incidence rate of 0.79/10^5^ (±0.74) for pIBD, 0.4/10^5^ (±0.42) for CD and 0.34/10^5^ (±0.4) for UC. From 2000 to 2020, pIBD incidence rose significantly, with an average annual increase of 12%. CD incidence showed a substantial upward trend, with a 13.1% annual increase, while UC incidence had a smaller, non-significant rise (5.6%). We considered stratifying incidence rates for age groups (but for many studied years, not all age groups were represented) and also for sex (but in the first decade, the number of cases was very low).

The north-west region of Romania is one of the eight development regions that correspond to the Nomenclature of Territorial Units for Statistics (NUTS) II-level divisions for European Union member states [55]. The six counties included in this region are Cluj, Sălaj, Bistrița-Năsăud, Maramureș, Satu Mare and Bihor. Of the six mentioned counties, Cluj-Napoca is the largest city and also the second largest Romanian city. The population of this region is estimated to be 12% of the total population [22]. The Emergency Clinical Hospital for Children (ECHC) serves as the only tertiary academic pediatric center in the north-west region and includes a pediatric gastroenterology department. In Romania, adult medical centers do not officially provide care for pediatric patients (under 18 years old). Therefore, only a very small proportion of children are probably diagnosed and treated by adult gastroenterologists. All children in our country are insured and have free access to the healthcare system; as such, disease monitoring in private medical facilities is rare. We consider that the vast majority of the pIBD cases in the north-west region were diagnosed and treated at the ECHC in Cluj-Napoca.

Kaplan et al. proposed a four-stage model to describe the evolution of IBD epidemiology: (1) emergence, (2) acceleration in incidence, (3) compounding prevalence and (4) prevalence equilibrium [11,23]. The third phase—compounding prevalence—describes the adult IBD situation in many Western world countries [17]. As for pIBD, most countries are in the emergence or acceleration in incidence phases. Evidence from Slovenia (nationwide study) and England (regional—Wessex) might suggest that they are entering into the compounding prevalence phase, in light of the plateauing trend seen in pIBD incidence [18].

While IBD is increasingly recognized as a global disease, its epidemiology in some regions, especially from medium–low and low-income countries, remains underexplored [20]. The lack of epidemiological data may be attributed to underdiagnosis due to limited healthcare infrastructure, diagnostic challenges, low disease awareness and under-reporting in the absence of national IBD registries [56]. Evaluating IBD incidence not only provides insights into disease emergence but also helps identify regional variations and potential environmental or genetic risk factors. Epidemiological data play a crucial role in improving early diagnosis, enabling timely intervention and refining disease management strategies for patients [18]. So far, information about IBD epidemiology in Romania was obtained only for the adult population from a multicenter prospective study conducted between June 2002 and June 2003; low incidence rates were observed for both CD and UC (0.5/10^5^ and 0.97/10^5^, respectively) [57]. Our results provide, for the first time, important insights into the incidence of pIBD and its increasing trend. The overall pIBD incidence in Romanian children currently appears to be among the lowest in the world.

In the first analyzed time frame (2000–2010), pIBD cases were scarce, with a very low average incidence rate of 0.3/10^5^ (±0.4), suggesting Romania to be in the first stage of the aforementioned model—emergence [11]. The north-west Region of Romania has direct borders with Hungary and almost one-fifth of the population from this region is Hungarian. Beyond geographical location, the two nations also share culinary traditions. Interestingly, pIBD incidence reported from the Hungarian Pediatric IBD Registry between 2007 and 2009 was 7.48/10^5^ [58]. Two other studies from the Hungarian Veszprem Province calculated an IBD incidence of 11.6/10^5^ (between 2002 and 2006 for patients under 20 years old) and 12.4/10^5^ (between 2007 and 2011 for those under 18 years of age) [29,59]. The explanation behind this great difference between IBD incidence in Romania and Hungary remains to be determined in the future and might concern genetic–environmental interactions. IBD incidence closer to the one from our study was observed in Tuzla Canton, Bosnia–Herzegovina, between 1995 and 2006, but for those under 14 years of age. Pavlovic et al. reported a CD incidence of 0.3/10^5^ and Salkic et al. reported a UC incidence of 0.2/10^5^, which would sum up an overall IBD incidence of 0.5/10^5^ [45,46]. Northern European countries are on the opposite side of our observations for an overlapping time period (2005–2009). Data obtained from the national Finnish registry reveal a high incidence of pIBD in those under 19 years of age (20.7/10^5^) [27]. Also, a high pIBD incidence of 18.9/10^5^ was observed in the IBD cohort of the Uppsala Region, Sweden [60,61]. In the year 2010, a pIBD incidence of 1.42/10^5^ (±0.5) was noted in our study, the highest in the aforementioned time frame, mainly due to the UC cases. For the same year, pIBD incidence in Moldova, a neighboring country, was 2.9/10^5^, as cited by Burisch, with information collected from a single medical center; the majority of cases were diagnosed as UC, with an incidence of 2.7/10^5^ [42].

For the next 10 years included in our study (2011–2020), the average pIBD incidence increased to 1.33/10^5^ (±0.66), probably bringing Romania into the second phase—acceleration in incidence—of the model described by Kaplan et al. [11]. Data for an overlapping time frame (2009–2019) from Tuzla Canton, Bosnia–Herzegovina, showed a slightly more elevated pIBD incidence of 2.49/10^5^ [53]. Northern European countries (Finland, Denmark, United Kingdom) and Germany maintained remarkably higher pIBD incidence rates compared to those from our study (22.7/10^5^, 16.4/10^5^, 9.37–12/10^5^ and 16.9/10^5^, respectively) [27,33,36,40,47]. The maximum pIBD and UC incidence rates in the north-west region of Romania were in 2015 (2.86/10^5^ for pIBD, 1.71/10^5^ for UC, 1.14/10^5^ for CD). Observations from the Italian pIBD Registry show incidence rates as high as 2.2/10^5^ in the 2009–2018 time interval, which are marginally lower than our findings [12].

Many studies have shown higher rates of pediatric CD compared to UC in Europe, North America and Oceania, except for Scandinavia (Finland), Northern California, Southern Europe (Italy, Croatia) and Eastern Europe (Poland), where the incidence of UC exceeds that of CD. The reasons for the differences in rates among the IBD subtypes are still unclear [19]. We observed that in the north-west region of Romania, pediatric CD was more frequent than UC overall and in the last studied decade (0.4/10^5^ vs. 0.34/10^5^, respectively, and 0.72/10^5^ vs. 0.51/10^5^). IBD-U cases were diagnosed starting in 2016 and were scarce, so we could not make any pertinent observations. When evaluating global time trends for pIBD incidence, a 2020 systematic review revealed that incidence rates increased, in at least some age and disease-specific subgroups, in the majority of the included studies [18]. In Europe, the ratio of pediatric CD to UC incidence rose significantly over time, although the rate has slightly declined during recent years [20]. A national study conducted in Poland between 2009 and 2020 analyzed data from the administrative healthcare database and observed a downward trend for the incidence of CD, with no differences when comparing particular age groups (over the years 2014–2018); also, it showed a constant trend for UC incidence in those under 20 years of age (between 2012 and 2018) [25].

Our findings mirror the positive temporal trend for pIBD and CD incidence. An Israeli nationwide IBD study, conducted between 2005 and 2017, showed that incidence in the pediatric group continued to increase, most significantly in the 10–18-years-old age group. Incidence rates for CD rose from 7.3 to 8.3/10^5^ (AAPC, +1.9%, *p* < 0.05), and for UC, they rose from 2.6 to 4.4/10^5^ (AAPC, +5.8%, *p* < 0.05). In comparison, in our study, we observed a greater increase in CD incidence rates (APC 13.1, 95% CI: 8–18.5, *p* < 0.001) and also an increase in UC incidence, but without statistical significance (APC 5.67, 95% CI: −1.5–13.4, *p* > 0.05). Two regional studies from the Czech Republic (Pilsen and South Moravia regions) showed similar high pIBD incidence rates (10/10^5^, respectively 9.8/10^5^) and both revealed a significant rise in pIBD and CD incidence over the 16-year study period (2000–2015, respectively, and 2002–2017) [62,63].

Over the past two decades, in Romania, there has been no major change in the population size or genetic profile, suggesting that the rising incidence of pIBD is un-likely to be driven by hereditary factors alone. In fact, genetic predisposition for IBD was observed in only a very small percentage of our cases (3.1% had a positive family history for IBD). Similarly, only five patients (5.3%) belonged to the very-early-onset IBD group (under six years old), where monogenic forms of IBD are more likely to occur. Recent studies have indicated that monogenic IBD can also manifest in adolescents and adults, accounting for over one-third of cases [64]. However, genetic testing is not a common practice and patient information in our study was collected retrospectively.

The significant socio-economic shift following the end of the communist era likely played a key role in the rising incidence of pIBD in Romania. The adoption of a Westernized lifestyle brought about particular changes in diet, characterized by increased consumption of processed foods, a higher intake of refined sugars, animal protein and fat, and decreased consumption of fiber-rich fruits and vegetables, along with a reduced intake of home-cooked meals. A study analyzing dietary habits among IBD patients in Romania and Belgium confirmed this eating pattern [65]. Processed foods contain various artificial additives, including emulsifiers (e.g., carboxymethylcellulose, polysorbate-80), sweeteners (e.g., aspartame, sucralose), stabilizers (e.g., carrageenan), preservatives and colorants. Numerous animal studies have demonstrated that these additives can disrupt gut barrier integrity, induce dysbiosis and promote inflammation [66,67,68]. A recent study from the GEM cohort provides new evidence supporting the role of gut microbiome composition in CD pathogenesis. The study identified a preclinical microbial signature associated with increased CD risk, characterized by a higher abundance of Ruminococcus torques and Blautia taxa, alongside reduced levels of Roseburia genus and Faecalibacterium, particularly Faecalibacterium prausnitzii. Additionally, the findings suggest that a decline in gut microbiome-derived anti-inflammatory metabolites may precede CD onset, further supporting the role of microbial dysbiosis in disease initiation [69]. Through these mechanisms, the consumption of a Western-style diet may contribute to the pathogenesis of IBD [35,70,71].

The strong influence of diet on disease development is further supported by the effectiveness of some nutritional interventions in reducing inflammation. EEN has been the first-line and standard dietary treatment for the induction of remission in pediatric patients with mild-to-moderate luminal CD. EEN provides essential nutritional support, promoting linear growth and bone health. However, EEN seems to be difficult to maintain, and the reintroduction of whole foods after six to eight weeks of treatment can be accompanied by the recurrence of inflammation and gastrointestinal symptoms [5,72]. CDED is a dietary intervention used in combination with partial enteral nutrition that excludes or limits dietary components that are potentially pro-inflammatory while encouraging whole, minimally processed foods. CDED showed high remission rates when used for the induction of remission, especially in mild-to-moderate CD cases, and provided adequate nutrient intake [73]. CDED been recently recommended by the ESPEN guideline on clinical nutrition in IBD [74]. The findings regarding the role of diet as maintenance therapy for CD remain inconclusive. In UC, evidence of diet therapy for both the induction and maintenance of remission is limited; however, emerging research suggests that plant-based diets, characterized by high fiber content and reduced animal protein intake, may offer therapeutic benefits [10]. Various dietary components (including probiotics, bovine immunoglobulin, vitamin D, omega-3 fatty acids, flavonoids, polyphenols, curcumin and phosphatidylcholine) and plant-derived substances (such as arjuna, soy protein and nettles) might play a role in supporting the treatment of IBD; a comprehensive review suggests that these components may help reduce oxidative stress, regulate immune responses and inhibit pro-inflammatory pathways, potentially improving treatment outcomes and maintaining remission in IBD patients [75].

Antibiotic use, especially at younger ages, was linked to the development of IBD [76,77]. Global data indicate a rising trend in antibiotic consumption, particularly in middle-income countries, including Romania [78]. In addition, other environmental factors that might have contributed to the increasing pIBD incidence in the north-west region of Romania include passive smoking, exposure to infections and prolonged non-steroidal anti-inflammatory drug consumption [79,80,81]. Data from recent cohort studies suggest that a larger family size in the first year of life and living with a dog between the ages of 5 and 15 can reduce the risk of developing IBD; also, living with a bird increases the risk of IBD [82]. In Romania, families have reduced in size after the end of the communist era and their pronatalist policies, and this is an aspect that might be taken into consideration when facing rising IBD incidence.

Additionally, improvements in healthcare infrastructure and diagnostic capabilities may have led to greater recognition of pIBD in our region. Over time, awareness among healthcare providers increased, advanced imaging and endoscopic techniques became more available and symptoms were identified earlier (at a younger age, in adolescents in the inflammatory stage) [18]. All of these factors have likely contributed to higher reported incidence rates. Although our study did not directly assess the role of these environmental and diagnostic factors due to the retrospective nature of data collection, their potential influence cannot be overlooked when interpreting the rising incidence of pIBD in Romania.

Our study comes with several inherent limitations. The first to mention is the retrospective manner used for data collection. Also, it was a single-center study, but in the explored area, ECHC is the only tertiary medical center able to diagnose and treat pIBD with experience and accuracy. As for primary care physicians, children with chronic digestive pathology from Romania are referred to a tertiary level. It could be argued that a proportion of patients may have been unrecruited because they were monitored by adult gastroenterologists, but this number would be very small (probably adolescents with mild IBD) since adult medical centers do not officially provide care for pediatric patients. Thirdly, even though we considered the revised PORTO criteria of ESPGHAN for diagnosis, we observed that some cases lacked small bowel imaging and upper gastrointestinal endoscopy, especially in the first decade. However, the endoscopic and histopathological aspects and distribution of lesions were consistent with the established diagnosis. The main role of this study, being the first of its kind, was to evaluate pIBD incidence in the north-west region of Romania, and the accuracy of the pIBD diagnostic is very high.

## 5. Conclusions

Overall, this study highlights the significant increase in the incidence of pIBD, especially CD, in the north-west region of Romania, emphasizing the importance of continued surveillance and research to understand the complex factors contributing to this rising incidence. With a low pIBD incidence compared to other countries in the world, Romania may be in the early stages of a broader trend that will require enhanced clinical care, improved diagnostic tools and greater awareness among healthcare providers to address the growing needs of children affected by IBD in the region.

## Figures and Tables

**Table 1:** Distribution of patients according to year of diagnosis, age group, type of disease and sex in north-west region of Romania (female—F, male—M, Crohn’s disease—CD, ulcerative colitis—UC, pediatric inflammatory bowel disease—pIBD).

	Age Group	0 to <6 Years		6 to <10 Years		10 to <18 Years				Age Group	0 to <6 Years		6 to <10 Years		10 to <18 Years				Age Group	0 to <6 Years		6 to <10 Years		10 to <18 Years	
Year	Disease	F	M	F	M	F	M		Year	Disease	F	M	F	M	F	M		Year	Disease	F	M	F	M	F	M
2000	CD	0	0	0	0	0	0		2007	CD	0	0	0	0	0	0		2014	CD	0	0	0	0	1	2
	UC	0	0	0	0	0	0			UC	0	0	0	0	0	0			UC	0	0	1	0	2	0
	pIBD	0	0	0	0	0	0			pIBD	0	0	0	0	0	0			pIBD	0	0	1	0	3	2
2001	CD	0	0	0	0	0	0		2008	CD	0	0	0	1	1	0		2015	CD	0	0	0	0	2	4
	UC	0	0	0	0	0	0			UC	0	0	0	0	0	0			UC	1	0	1	0	2	5
	pIBD	0	0	0	0	0	0			pIBD	0	0	0	1	1	0			pIBD	1	0	1	0	4	9
2002	CD	0	0	0	0	1	0		2009	CD	0	0	0	0	2	0		2016	CD	0	0	0	1	2	3
	UC	0	0	0	0	0	0			UC	0	0	0	0	1	0			UC	0	0	0	0	0	0
	pIBD	0	0	0	0	1	0			pIBD	0	0	0	0	3	0			pIBD	0	1	0	1	2	3
2003	CD	0	0	0	0	0	0		2010	CD	0	0	0	0	1	1		2017	CD	0	0	1	0	2	1
	UC	1	0	0	0	1	1			UC	0	0	0	2	0	4			UC	0	0	0	0	2	1
	pIBD	1	0	0	0	1	1			pIBD	0	0	0	2	1	5			pIBD	0	0	1	0	4	4
2004	CD	0	0	0	0	0	0		2011	CD	0	0	0	0	0	1		2018	CD	0	0	0	0	3	0
	UC	0	0	0	0	1	0			UC	0	0	0	0	0	1			UC	0	0	0	0	1	1
	pIBD	0	0	0	0	1	0			pIBD	0	0	0	0	1	2			pIBD	0	0	0	0	4	1
2005	CD	0	0	0	0	0	0		2012	CD	0	0	0	0	2	2		2019	CD	0	0	0	0	0	3
	UC	0	0	0	0	1	0			UC	0	0	1	0	0	0			UC	0	0	1	1	1	1
	pIBD	0	0	0	0	1	0			pIBD	0	0	1	0	2	2			pIBD	0	0	1	1	2	4
2006	CD	0	0	0	0	0	1		2013	CD	0	0	0	0	0	2		2020	CD	0	0	0	0	0	8
	UC	0	0	0	0	0	1			UC	1	0	0	0	1	0			UC	0	0	0	0	2	0
	pIBD	0	0	0	0	0	2			pIBD	1	0	0	0	1	2			pIBD	0	1	0	0	2	8

**Table 2:** Epidemiological data for pIBD in the north-west region of Romania during 2000–2020.

Year	Cases	Population	Crude Rate (CR)	Age-Adjusted Rate (AAR)	Standard Error (SE)	Lower 95% CI	Upper 95% CI
2000	0	673,805	0	0	0.01	−0.02	0.02
2001	0	664,082	0	0	0.01	−0.02	0.02
2002	1	650,872	0.15	0.13	0.13	−0.12	0.38
2003	3	635,385	0.47	0.43	0.25	−0.06	0.92
2004	1	620,138	0.16	0.14	0.14	−0.13	0.41
2005	1	603,003	0.17	0.16	0.16	−0.15	0.47
2006	2	587,201	0.34	0.29	0.21	−0.12	0.70
2007	0	570,654	0	0	0.01	−0.02	0.02
2008	2	554,887	0.36	0.32	0.23	−0.13	0.77
2009	3	547,296	0.55	0.48	0.28	−0.07	1.03
2010	8	541,912	1.48	1.4	0.49	0.44	2.36
2011	3	537,399	0.56	0.53	0.31	−0.08	1.14
2012	5	531,879	0.94	0.9	0.4	0.12	1.68
2013	4	528,155	0.76	0.73	0.36	0.02	1.44
2014	6	524,351	1.14	1.09	0.45	0.21	1.97
2015	15	524,824	2.86	2.73	0.71	1.34	4.12
2016	7	523,828	1.34	1.26	0.48	0.32	2.20
2017	9	522,578	1.72	1.64	0.55	0.56	2.72
2018	5	521,722	0.96	0.91	0.41	0.11	1.71
2019	8	519,134	1.54	1.47	0.52	0.45	2.49
2020	11	515,232	2.13	2.01	0.61	0.81	3.21

**Table 3:** Epidemiological data for pediatric CD in the north-west region of Romania during 2000–2020.

Year	Cases	Population	Crude Rate (CR)	Age-Adjusted Rate (AAR)	Standard Error (SE)	Lower 95%CI	Upper 95%CI
2000	0	673,805	0	0	0.01	−0.02	0.02
2001	0	664,082	0	0	0.01	−0.02	0.02
2002	0	650,872	0	0	0.01	−0.02	0.02
2003	3	635,385	0.47	0.43	0.25	−0.06	0.92
2004	1	620,138	0.16	0.14	0.14	−0.13	0.41
2005	1	603,003	0.17	0.16	0.16	−0.15	0.47
2006	1	587,201	0.17	0.17	0.16	−0.14	0.48
2007	0	570,654	0	0	0.01	−0.02	0.02
2008	0	554,887	0	0	0.01	−0.02	0.02
2009	1	547,296	0.18	0.15	0.15	−0.14	0.44
2010	6	541,912	1.11	1.05	0.43	0.21	1.89
2011	2	537,399	0.37	0.35	0.25	−0.14	0.84
2012	1	531,879	0.19	0.18	0.18	−0.17	0.53
2013	2	528,155	0.38	0.36	0.26	−0.15	0.87
2014	3	524,351	0.57	0.54	0.31	−0.07	1.15
2015	9	524,824	1.71	1.63	0.54	0.57	2.69
2016	0	523,828	0.00	0	0.01	−0.02	0.02
2017	3	522,578	0.57	0.55	0.32	−0.08	1.18
2018	2	521,722	0.38	0.37	0.26	−0.14	0.88
2019	4	519,134	0.77	0.75	0.37	0.02	1.48
2020	2	515,232	0.39	0.37	0.26	−0.14	0.88

**Table 4:** Epidemiological data for UC in the north-west region of Romania during 2000–2020.

Year	Cases	Population	Crude Rate (CR)	Age-Adjusted Rate (AAR)	Standard Error (SE)	Lower 95%CI	Upper 95%CI
2000	0	673,805	0	0	0.01	−0.02	0.02
2001	0	664,082	0	0	0.01	−0.02	0.02
2002	0	650,872	0	0	0.01	−0.02	0.02
2003	3	635,385	0.47	0.43	0.25	−0.06	0.92
2004	1	620,138	0.16	0.14	0.14	−0.13	0.41
2005	1	603,003	0.17	0.16	0.16	−0.15	0.47
2006	1	587,201	0.17	0.17	0.16	−0.14	0.48
2007	0	570,654	0	0	0.01	−0.02	0.02
2008	0	554,887	0	0	0.01	−0.02	0.02
2009	1	547,296	0.18	0.15	0.15	−0.14	0.44
2010	6	541,912	1.11	1.05	0.43	0.21	1.89
2011	2	537,399	0.37	0.35	0.25	−0.14	0.84
2012	1	531,879	0.19	0.18	0.18	−0.17	0.53
2013	2	528,155	0.38	0.36	0.26	−0.15	0.87
2014	3	524,351	0.57	0.54	0.31	−0.07	1.15
2015	9	524,824	1.71	1.63	0.54	0.57	2.69
2016	0	523,828	0.00	0	0.01	−0.02	0.02
2017	3	522,578	0.57	0.55	0.32	−0.08	1.18
2018	2	521,722	0.38	0.37	0.26	−0.14	0.88
2019	4	519,134	0.77	0.75	0.37	0.02	1.48
2020	2	515,232	0.39	0.37	0.26	−0.14	0.88

## Data Availability

The original contributions presented in this study are included in the article; further inquiries can be directed to the corresponding author, within the legal regulations.
